# Effects of back exercises versus transcutaneous electric nerve stimulation on relief of pain and disability in operating room nurses with chronic non-specific LBP: a randomized clinical trial

**DOI:** 10.1186/s12891-022-05227-7

**Published:** 2022-03-26

**Authors:** Fereshteh Jalalvandi, Reza Ghasemi, Maryam Mirzaei, MohammadBagher Shamsi

**Affiliations:** 1 Department of Operating Room, School of Paramedical, Kermanshah University of Medical Sciences, Kermanshah, Iran; 2 Department of Physiotherapy, School of Rehabilitation Sciences, Kermanshah University of Medical Sciences, Kermanshah, Iran

**Keywords:** Exercise therapy, Transcutaneous electric nerve stimulation, Low Back pain

## Abstract

**Background:**

Low back pain (LBP) is one of the most common musculoskeletal disorders related to working. Due to the nature of nursing work, this problem is often seen in nurses, including those who work in the operating rooms. Depending on the cause, there are various surgical and non-surgical methods to treat LBP. The present study was aimed to compare the effect of two therapeutic methods of back exercises and transcutaneous electrical nerve stimulation (TENS) on the disability and pain of operating room nurses with LBP.

**Methods:**

In this clinical trial forty-four eligible operating room nurses (30 women, 14 men, mean age: 37.86 ± 6.74) with chronic nonspecific LBP were randomly assigned to back exercises (including the strengthening and stretching exercise (*n* = 22)) or TENS (*n* = 22) groups by permuted block randomization method.

These interventions were performed in both groups three sessions of 15 min per week for 6 weeks. The McGill pain questionnaire for back pain and the Oswestry disability questionnaire for disability assessment were completed immediately before and after the interventions.

**Results:**

After 6 weeks, the mean of pain and disability decreased significantly in both groups compared to the baseline. Based on the results, significant decreases in the pain score (mean difference (95% CI): − 8.95 (− 12.77 to − 5.14); *P*-value < 0.001) and disability score (mean difference (95% CI): − 8.73(− 12.42 to − 5.03); *P*-value < 0.001) were revealed in the back exercises group after the intervention compared to the baseline. In addition, after the intervention in TENS group, the mean pain intensity and disability showed significant decrease, respectively (mean difference (95% CI): − 16.18 (− 19.81 to − 12.55); *P*-value < 0.001; mean difference (95% CI): − 15.82 (− 19.24 to − 12.40); *P*-value < 0.001).

After adjusting for the baseline values, the TENS group had a significantly higher pain score reduction than the back exercises group (mean difference (95% CI): − 4.23 (− 8.03 to − 0.44); *P*-value =0.030; Cohen’s d = 0.81). In addition, TENS led to a significant more decrease in the disability scores compared to the back exercises (mean difference (95% CI): − 3.99 (− 7.35 to − 0.64); *P*-value =0.021; Cohen’s d = 0.73). Furthermore, a statistically significant time by group interaction effect on pain and disability score was found (interaction *p* < 0.001).

**Conclusion:**

Pain and disability were improved in both groups following 18 intervention sessions. However, pain and disability were improved to a greater extent in the TENS group than in the back exercises group.

**Trial registration:**

The trial was retrospectively registered in the Iranian Registry of Clinical Trials (www.irct.ir) on 03/02/2019 as IRCT20180408039227N1.

## Background

Musculoskeletal disorders (MSDs) are major public health problems worldwide [1]. The high outbreak, health problems and economic burden caused by MSDs in recent decades have led to the emergence of these disorders as an important dilemma in the health of communities [2]. MSDs are combinations of painful disorders of muscles, tendons, joints and nerves that can affect all parts of the body, especially the neck, upper limb and back [3]. Low back pain (LBP) is one of the most common MSDs related to working and one of the most important [4, 5]. LBP is the leading cause of disability in the world and according to 2010 reports, 21.7 million people annually suffer from this disability [6]. The outbreak of LBP is 47% in the United States and 40–60% in Asian countries [4], and this problem is one of the main health problems in healthcare workers [7]. LBP is a complex disorder that is associated with many unpleasant consequences such as physical disability, psychosocial disorder and increased use of health care resources [8]. Despite significant advances in the prevention and treatment of spinal problems, LBP is still one of the most common musculoskeletal issues in various communities [9].

There are various non-surgical and surgical methods in the field of treatment and relief of LBP, depending on its cause. Pharmacological methods are usually only symptomatic treatment and do not eliminate the cause of the disease and are associated with complications such as drug dependence, overdose and abuse. Today, the use of non-pharmacological methods to control pain has received more attention and is progressing. Transcutaneous electrical nerve stimulation (TENS) is a non-pharmacological method of pain relief [10]. The advantages of this method are its safety, non-invasiveness, and harmlessness [11]. On the other hand, most clinical guidelines prescribe the use of exercise to treat chronic LBP, although there is no consensus on the preference of one exercise over the other [12]. Meanwhile, various studies show that exercises such as William’s exercise, which is one type of therapeutic exercise useful for LBP that improves the activity of gluteal and abdominal muscles, have been effective on nurses’ LBP [13]. Therefore, in this case, comparing different treatment methods can be useful.

Despite the variety of different methods for relieving LBP, no study was found to compare exercise and TENS on chronic LBP among medical personnel. Nurses as part of healthcare professionals who have an important role in patient care, due to their hard work, are the subjects of suffering LBP. In the operating room and nursing duties, conditions such as lifting patients, sitting and standing for long periods, excessive rotations of the torso, pushing or lifting heavy objects and equipment during surgery, are usual. Nursing is the most leading occupation in the prevalence of LBP and injury [14, 15]. The results of a meta-analysis indicated that the prevalence of LBP in nurses was 76.0% (95% CI, 69.0–81.8%) [15]. Therefore, the present study aimed to determine and compare the effects of exercise and TENS on chronic LBP of the operating room nurses (and nurse aides) of Imam Reza Hospital in Kermanshah, Iran.

## Methods

### Study design

This single-blind randomized clinical trial with parallel group’s design was performed in Imam Reza (AS) hospital in Kermanshah, IRAN from 04 February 2019 (first assessment) to 20 March 2019 (last assessment). After obtaining permission from the ethics committee of Kermanshah University of Medical Sciences (Reference number: IR.KUMS.REC.1397.815) and registration in the Iranian Registry of Clinical Trials (IRCT) database (Reference number: IRCT20180408039227N1 on 03/02/2019), all methods were performed in accordance with the Declaration of Helsinki. Furthermore, the trial was reported based on the Guidelines for Consolidated Standards of Reporting Trials (CONSORT).

### Participants

**Fig. 1:**
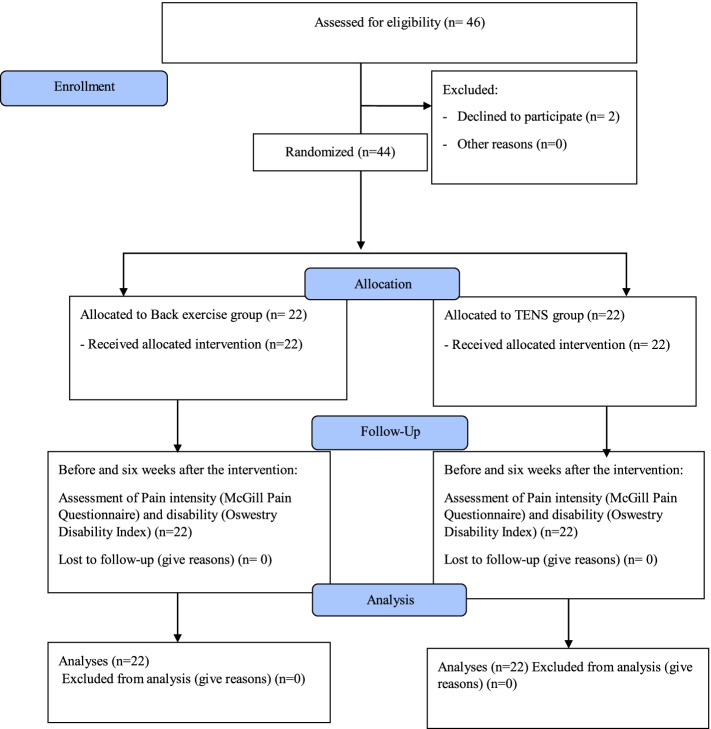
Flow diagram of the study

Among the nurse staff and nurse aides working in the operating room of Imam Reza Hospital, forty-four participants (30 women, 14 men, mean age: 37.86 ± 6.74) with chronic non-specific LBP were selected based on their history and examination by an experienced physiotherapist. The inclusion criteria were having at least 3 months of LBP, no history of lumbar surgery or spinal fractures, pregnancy, rheumatic diseases, history of cancer, pacemaker, severe deformity, no congenital abnormalities of the spine and anti-inflammatory and analgesic drug used during the study, In addition, unwillingness to continue to participate in the study was considered as an exclusion criterion. All participants gave written informed consent before entering the trial. Using permuted block randomization method with a block size of four, they were randomly assigned to two groups: back exercises (*n* = 22), or TENS (*n* = 22) groups (Fig. 1). In order to control the potential selection bias, the random allocation sequence was generated by a person who was not involved in the enrollment or screening of participants. The statistician who analyzed the data was blind to the group allocation of participants. Because of the nature of interventions (using an instrument in one group), blinding the participants was not possible. In addition, the researchers that provided the treatments for participants were not blind, because they were involved in the intervention process and data collection.

Based on the reduction in the pain scores according to a previous study (Mean difference (95% CI) = − 1.2 (− 2 to − 0.4) and − 0.1 (− 0.1 to 0.7), respectively for TENS group and control group), the required sample size was estimated. A 95% confidence level and a power of 80% to be 22 for each group were considered [16].

### Interventions

After selecting the samples, participants were informed about this study, how to perform the intervention, the duration and the place of the study. The training of the samples was done in the first session. The length of times of applying the two types of intervention, including exercises and TENS were equal. It was 15 min. The interventions were performed three sessions every week for 6 weeks in the workplace of the participants (in the restroom of the hospital operating room ward), so there was no need to leave the work.

**Fig. 2:**
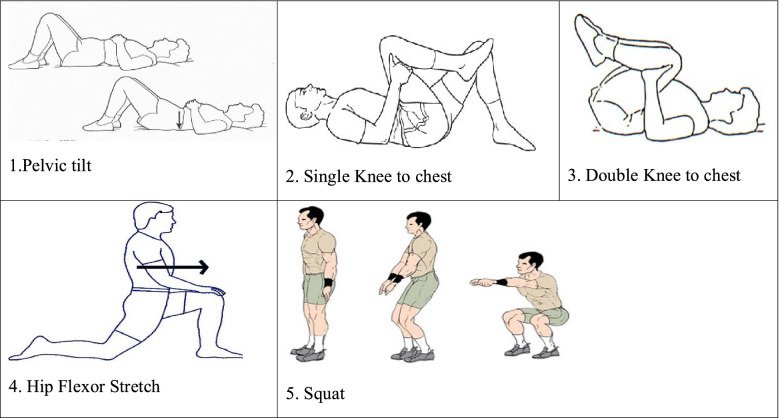
Back exercises

Also, the back exercises group performed exercises for strengthening and stretching of the back and pelvis muscles which included 1) Pelvic tilt 2) Single knee to chest 3) double knee to chest 4) hip flexor stretches 5) squat (Fig. 2) [17]. In each set, these exercises were repeated 10–12 times. They did as many sets of exercise as up to 15 min.

In the TENS group, the Trio 300 dual-channel TENS device from ITO. Co was used. The electrodes of the device were placed in the lower back region. A frequency of 100 Hz, duration of 0.2 ms and an intensity that increased as the participants felt a comfortable prickling sensation (about 15 mA) was applied [18].

### Primary outcome measures

Before the intervention and at the end of the sixth week (Immediately after the intervention), the McGill pain questionnaire and the Oswestry questionnaire were completed to assess the pain intensity and performance of the participants, respectively.

### Pain

The pain was assessed by the short-form of McGill Pain Questionnaire (MPQ***).*** This questionnaire has 20 items, and its purpose is to assess the subject’s perception of pain in different aspects (four aspects of sensory perception of pain, affective emotional perception, evaluative perception and perception of diverse pain (Miscellaneous)) [19]. Internal consistency was found by Cronbach’s alpha to be 0.76 for total scores. Also, the reliability of this questionnaire has been assessed and confirmed in previous studies (intraclass correlation coefficient (ICC) for total SF-MPQ ranged from 0.90 to 0.94 and Cronbach’s alpha ranged from 0.84 to 0.91) [20–22].

### Disability

To assess the disability, the Persian version of the Oswestry Disability Index (ODI) was administered. It is a scale with a range from 0 to 100 describing the level of disability about pain intensity, sexual function, sleep quality, and level of performance in daily activities such as sit, walk, lift, stand, work, and travel. The reliability of the Persian version of this questionnaire has been tested in this current study (Cronbach’s alpha = 0.84) and approved in a study by Mousavi, et al. (Cronbach’s alpha = 0.83; ICC = 0.91) [23].

### Statistical analysis

Statistical analyses were performed using the IBM SPSS statistics software (version 23) and for all statistical tests*, p* < .05 was set as the significant level. Mean ± SD and frequency (percentage) were used to express the continuous and categorical variables, respectively. According to the results of the Shapiro-Wilk test, all variables of the study had a normal distribution. Differences in general characteristics of the participants between the two groups were analyzed using the independent t-test and chi-square test.

A mixed ANOVA test was conducted as follows: (1) the main effect of groups (back exercises vs. TENS) was determined by any conspicuous difference in pain and disability observed between two groups; (2) the main effect of time (pre vs. post) was determined by any conspicuous difference in pain and disability observed amidst the two different time points; (3) and the time × group (interaction) effect.

In this regard, to assess the time effect within each group separate repeated measure ANOVA tests were performed. Thereafter, a 2 × 2 (time × Group) ANOVA with repeated measures on time was conducted to assess potential interaction. Also, Cohen’s d effect sizes were determined by dividing the difference between the means by the standard deviation. The values of 0.2, 0.5, and 0.8 were regarded as small, moderate, and large effect sizes, respectively [24]. *P*-values less than 0.05 were considered statistically significant for all of these tests.

## Results

All enrolled participants (*n* = 44) completed every intervention sessions (*n* = 22) and no adverse events were reported (Fig. 1).

### Demographic data

As shown in Table 1, the general characteristics of the participants were not significantly different between the back exercises and TENS groups (all *p*-values> 0.05). In addition, all baseline measurements of the two groups indicated no significant differences between them with respect to pain scores (*p*-value = 0.93), and disability scores (*p*-value = 0.59).

**Table 1:** Baseline characteristics of the participants in each group

Groups	Category	Back exercises group (***n*** = 22)	TENS group (***n*** = 22)	***p***-value
**Age (year)(Mean** ± **SD)**		37.86 ± 6.74	36.05 ± 5.11	0.319^**#**^
**Sex**	**Female**	16 (72.7%)	14 (63.6%)	0.517^*^
	**Male**	6 (27. 3%)	8 (36.4%)	

*TENS* Transcutaneous Electric Nerve Stimulation/

Numerical data are presented as mean ± SD and categorical variables are presented as number (%)/ ^#^Based on independent samples t-test/ *Based on chi-square test

### Primary outcome measures

After 6 weeks of intervention, the mean pain and disability scores decreased significantly in both groups compared to baseline. Based on the repeated measure analysis of variance, significant decreases in the pain score (mean difference (95% CI): − 8.95 (− 12.77 to − 5.14); *p*-value < 0.001) and disability score (mean difference (95% CI): − 8.73(− 12.42 to − 5.03); *p*-value < 0.001) were revealed in the back exercises group after the intervention compared to the baseline values. In addition, after the intervention in TENS group, the mean pain intensity and disability score showed a significant decrease, respectively (mean difference (95% CI): − 16.18 (− 19.81 to − 12.55); *p*-value < 0.001; mean difference (95% CI): − 15.82 (− 19.24 to − 12.40); *p*-value < 0.001).

At the end of the study, the TENS group presented significantly more pain score reduction than the back exercises group (mean difference (95% CI): − 4.23 (− 8.03 to − 0.44); *p*-value =0.030; Cohen’s d = 0.81). In addition, TENS led to a significant more decrease in the disability scores compared to the back exercises (mean difference (95% CI): − 3.99 (− 7.35 to − 0.64); *p*-value =0.021; Cohen’s d = 0.73) (Table 2).

**Table 2:** Comparison of pain and disability scores among the two groups of study (Back exercise and TENS group)

Variables	Measurement period	Back exercises group (***n*** = 22)	TENS group (***n*** = 22)	Main effect of group	Interaction effect between time and group
Pain score	Baseline	46.91 ± 9.09	51.36 ± 8.46	*p*-value =0.030; F = 5.06; df = 1 MD (95% CI): −4.23 (−8.03 to −0.44)	*p*-value =0.007; F = 8.15; df = 1
	After intervention	37.95 ± 8.21	35.18 ± 4.49		
	MD (95% CI)	−8.95(−12.77 to −5.14)	−16.18(−19.81 to − 12.55)		
	Main effect of time	*p*-value < 0.001; F = 24.1; df = 42	*p*-value < 0.001; F = 102.8; df = 42		
Disability score	Baseline	23.10 ± 11.44	27.91 ± 6.85	*p*-value =0.021; F = 5.74; df = 1; MD (95% CI): −3.99 (−7.35 to −0.64)	*p*-value =0.001; F = 8.57; df = 1
	After intervention	14.36 ± 6.55	12.09 ± 5.93		
	MD (95% CI)	−8.73(−12.42 to −5.03);	−15.82(−19.24 to − 12.40)		
	Main effect of time	*p*-value < 0.001; F = 23.8; df = 42	*p*-value < 0.001; F = 86.1; df = 42		

Mean ± Standard deviation was reported/ MD (95% CI): Mean difference and 95% confidence interval/

**Fig. 3:**
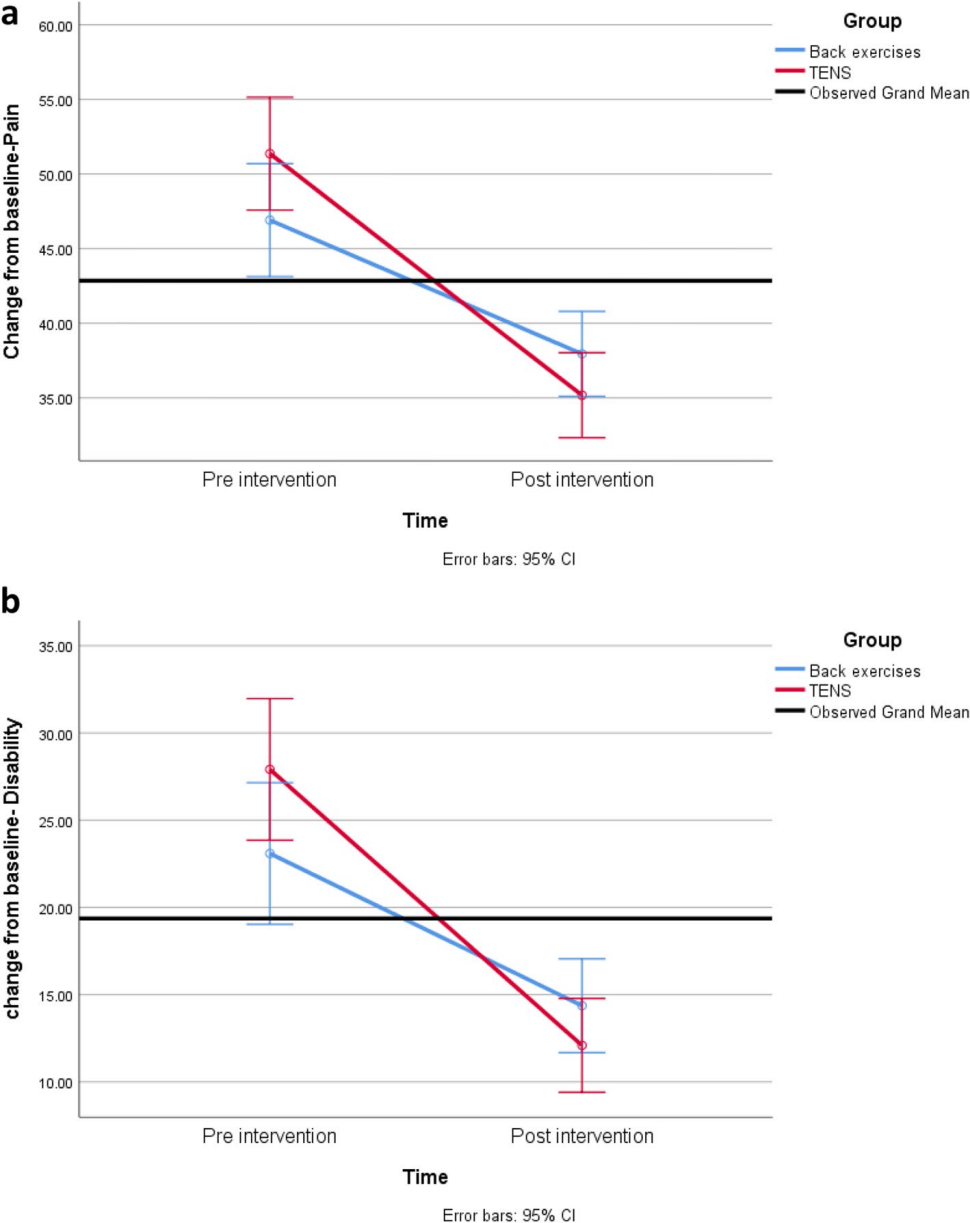
**a** Change from baseline in pain scores over time; **b** Change from baseline in disability scores over time

In addition, a statistically significant time by group interaction effect on pain and disability score was found (interaction *p* < 0.001) (Fig. 3).

## Discussion

The present study was aimed to compare the effect of back exercises and TENS on hospital operating room nursing staff suffering from chronic non-specific LBP. Based on the results of the study a significant more reduction in pain and disability scores was observed in the TENS group compared to the back exercises (all, *p* < 0.05), with outcome measures showing a large effect size (effect size ranged over = 0.73 to 0.81).

In a 1990 study by Deyo et al. on patients with LBP, the effect of stretching exercise and TENS on patients’ pain intensity measured with VAS was compared. The authors found that TENS had no greater effect than exercises [25]. In contrast to the previous study, the disability variable, which in this study was assessed with the Oswestry disability score, improved more in the TENS than in the exercises group [26], which is in line with our study.

In another study that SW Hahn et al. performed on patients who underwent coronary angiography and were in a supine position for a long time and so they suffered from LBP [27]. In one group exercise with TENS and in the other only exercise was applied. The results of this study showed that the rate of reduction of pain in the exercise plus TENS group was significantly higher than the only TENS group. This study did not have a control group in which only exercise was applied and could compare exercise independently to TENS [27]. Adding TENS to exercise could justify further improvement in this study (As applying two different factors is more effective and TENS stimulate large-diameter non-noxious afferents (A-beta) that subsequently reduces pain and makes the tissues ready to be more affected by exercise) [28].

Westcott et al. conducted a study on patients with LBP and examined the effects of exercise in a group as well as an exercise plus electrical stimulation in another group on pain [29]. This study could not show the superiority of either of these two interventions over the other. This study did not have a group in which only TENS was applied and TENS could be independently compared with exercise therapy [29].

Zerish et al. conducted another similar study in which patients with nonspecific LBP were divided into three groups. In one group only TENS was applied, in the second one only exercise and in the third, both TENS and exercise were applied. In this study, although both interventions led to reducing pain, neither was significantly different from the other and it showed no preference for either. Of course, the third group, which applied both interventions simultaneously, had a significant difference with other interventions [30, 31], which again, the combination of the two interventions could justify the greater impact.

A systematic review and meta-analysis study was performed by Resende et al. on the effect of TENS on patients with LBP and neck pain. Their results showed that electrical stimulation was beneficial in reducing LBP and neck pain. On the other hand, this study showed that the effect of TENS is a time-dependent variable and the evaluation time is important for the judgment. In general, in this study, no study reported the presence of pain during TENS application, but more research is needed in this field [31]. The results of these studies are consistent with our present study. However, in this study, a questionnaire was used, and because the questionnaires are self-reporting and subjective, patients may make mistakes or be biased in answering the questions.

Various therapeutic interventions have been introduced to treat LBP. One of these interventions is the therapeutic exercise, which is widely used in the treatment of LBP. Therapeutic exercise includes various interventions such as the aerobic exercise, the strengthening exercise and the stretching exercise for increasing flexibility. There is much evidence that exercise is effective in treating chronic LBP [32]. According to clinical practice guidelines, there is evidence for the effect of exercise for LBP in the acute and chronic stage and also after related surgeries, but these guidelines have not provided evidence for the prescription of TENS in this problem [33].

It is believed that conventional TENS in the segmental level by activating large-diameter Aβ fibers (induced by electrical stimulation), causes activation of the inhibitory interneurons in the dorsal horn of the spinal cord, so it reduces the firing rate of the projection neurons [34]. In addition, according to the gate control theory of pain that is proposed by Melzack and Wall, non-nociceptive inputs close the nerve “gates” to pain inputs, which blocks nociceptive impulses from passing to the central nervous system [34].

As it can be observed, there is a disagreement about the preference of exercise or TENS in reducing LBP. Part of the difference in the results of the studies of these two interventions can be due to the difference in the type of study design, sample size and in the case of TENS intervention due to the difference in stimulation mode and features, method of TENS application, and treatment duration and in terms of exercise due to differences in the type of exercise, its intensity and duration [26].

Chronic low back pain has a multidimensional nature, with biological, psychological, and social factors playing a role in the development and maintenance of this condition. TENS may have a positive effect on LBP; however, the effect is only immediate or short-term. However, therapeutic exercises have a basic and structural effect and cause lasting effects on the body. Perhaps the differences in the results of different studies can be attributed to this issue. Therefore, though both interventions are effective and can in turn cause beneficial changes in anybody, focusing on passive treatments will not help patients deal with the condition in the long term. The positive effect of TENS should be seen as an adjunct to other evidence-based clinically relevant treatments. Future studies should investigate the effect of other interventions and modalities to treat LBP in nurses.

### Study limitations

This is a single-center trial study, so selecting samples from just one hospital ward and the small number of participants in each group was problematic. Subsequent studies should include larger sample size, select participants from difference medical centers (hospitals) and from different job fields. . Due to some limitations, we did not include a control group receiving no intervention assigned as the “true control group.” Therefore, future trials should try to include a control group – if available. Since the nature of one of our interventions (TENS) was such that should use an instrument, therefore, it was impossible for the study to be blind for the participants.

## Conclusion

Pain and disability were improved in both groups following 18 intervention sessions. However, improvements in pain and disability were greater in the TENS group as compared to back exercises group. Therefore, our findings suggest that including a TENS component to rehabilitation program for operating room nurses with chronic non-specific LBP may be beneficial.

## Data Availability

The data collected and analysed in the present study are not publicly available due to ethical restrictions but are available from the corresponding author upon request.

## Abbreviations

ANCOVA: Analysis of Covariance
LBP: Low back pain
MPQ: McGill Pain questionnaire
MSDs: Musculoskeletal disorders
TENS: Transcutaneous electrical nerve stimulation
